# A Novel Association between Oxybutynin Use and Bilateral Acute Angle Closure Glaucoma: A Case Report and Literature Review

**DOI:** 10.7759/cureus.2732

**Published:** 2018-06-04

**Authors:** Abdullah Haddad, Mais Arwani, Osama Sabbagh

**Affiliations:** 1 Internal Medicine, Saint Clair Memorial Hospital; 2 Internal Medicine, Allegheny General Hospital - Western Pennsylvania Hospital Medical Education Consortium, Pittsburgh , USA; 3 Department of Ophthalmology, George Washington University, Washington , USA

**Keywords:** acute angle closure glaucoma, blurry vision, oxybutynin

## Abstract

We are reporting a case of a 62-year-old male presenting with headache and blurry vision. His condition resolved with cessation of the presumed offending medication and urgent bilateral laser peripheral iridotomies since he failed medical therapy. This case presents a novel association between oxybutynin and bilateral acute angle-closure glaucoma (AACG).

## Introduction

Acute angle-closure glaucoma (AACG) is characterized by narrowing or closure of the anterior chamber angle of the eye. Patients present clinically with decreased vision, halos around lights, headache, severe eye pain, and vomiting. By 2020, it is estimated that 79.6 million people will be affected by glaucoma, both open and closed angle, comprising the second leading cause of blindness worldwide [1]. Medications were found to be among the secondary causative for this condition.

## Case presentation

A 62-year-old Caucasian male with a history of type 2 diabetes mellitus and hypertension presented to the emergency room with acute onset blurry vision and headache. The patient was in his usual state of health until a few hours prior to his presentation. He was working on a presentation in a poorly lit room when symptoms started. His headache was frontal, sharp in quality, with no radiation, and was accompanied with blurry vision. There was no associated fever, chills, sinus congestion, focal weakness or numbness, head trauma, neck pain, jaw claudication, recent sick contact or travel. The patient described his vision blurriness as “glazed vision”. His last dilated eye examination was performed a month prior to his presentation and was found to be normal. Upon arrival to the emergency room, he started to complain of photophobia.

His primary care physician, a week prior to his presentation, started the patient on a daily 5 milligrams of extended-release oxybutynin. His other home medications included metoprolol, levothyroxine, metformin, and aspirin.

On physical examination, the pupils were mid-dilated, fixed and non-reactive to light or accommodation, 3.5 mm oculus dexter (OD) and 4.0 mm oculus sinister (OS). Upon visual acuity assessment, the patient was able to count fingers at four feet in the right eye and two feet in the left eye.

Computed tomography (CT) of the head was negative for acute intracranial hemorrhage or any other acute changes. Erythrocyte sedimentation rate (ESR) and C-reactive protein (CRP) levels were within normal laboratory limits. Ophthalmology consultation was obtained.

Slit lamp examination showed grade 2 epithelial and stromal corneal edema oculus uterque (OU) with some epithelial bullae OD. The examination also showed bilateral grade 2 to 3 perilimbal conjunctival injection, the irises were within normal limits, and the lens showed grade 1 nuclear sclerosis. Goldman intra-ocular pressure (IOP) was 55 OD and 56 OS while Tonopen was 63 OD and 65 OS. The posterior pole was poorly visualized due to corneal edema but the optic nerves looked perfused with no evidence of hemorrhage. The diagnosis of AACG was made and the patient was immediately treated with topical prednisolone 1%, pilocarpine 1%, bimatoprost 0.01%, and brimonidine-timolol 0.2%-0.5% ophthalmic solutions.

After 45 minutes and several cycles of eye drops use, IOP using Tonopen decreased to 55 OD and 56 OS. The patient started to notice improvement in visual changes as well. Visual acuity was 20/400 to counting fingers OU. Subsequently, the patient required urgent bilateral laser peripheral iridotomies.

Due to the recent normal eye examination and the timeline of the events, oxybutynin was believed to be the trigger of the event and it was discontinued. A follow-up with the patient a week later revealed normal visual acuity and normalization of IOP.

## Discussion

The aqueous humor is produced by the ciliary body and it flows from the posterior chamber through the pupil to the anterior chamber. It then flows through the trabecular meshwork to reach the venous circulation. Glaucoma is an ocular disease caused by the imbalance between aqueous humor production and drainage leading to optic neuropathy and vision loss should left untreated. Average age of presentation is 60 with 4:1 incidence ratio in women versus men [2].

Primary acute angle-closure typically presents in older, hyperopic patients while secondary angle-closure results when the anterior chamber angle becomes occluded as a result of conditions that push the iris or ciliary body forward. This is seen in the pupillary block mechanism that happens due to the iris margin coming in contact with the lens capsule preventing drainage of aqueous through the pupil, which exacerbates the anterior bowing of the iris. Patients may experience symptoms of headache, eye pain, halos around light, decreased vision, nausea, and vomiting. The outcomes of patients with acute angle-closure glaucoma depend on how early the disease is detected and treated. Because glaucoma damage to the optic nerve is generally not reversible and can occur within a matter of hours in the case of an acute angle-closure attack, it is important that an ophthalmologist evaluate the patients urgently to provide prompt diagnosis and treatment.

Medications such as topical anticholinergic, sympathomimetic dilating drops, tricyclic antidepressants, selective serotonin reuptake inhibitor (SSRIs), monoamine oxidase inhibitors, antihistamines, antiparkinsonian drugs, antipsychotic medications, and antispasmolytic agents have been reported to cause secondary AACG [3-6]. Hyponatremia caused by hydrochlorothiazide use was also reported to cause this condition [7].

Oral anticholinergic therapy is the mainstay of treatment in overactive bladder and urinary urge incontinence. This class of medication inhibits muscarinic receptors on detrusor smooth muscles fibers in the urinary bladder. The presence of muscarinic receptors elsewhere, such as salivary glands, eyes, and intestines, might lead to side effects as dry mouth, constipation, and in rare cases, AACG.

Altan‐Yaycioglu et al. reported no increase in intra-ocular pressure in 24 patients treated with oxybutynin for 28 days [8]. However, Sung and Corridan reported a case of a patient who developed unilateral AACG in the setting of oxybutynin treatment [9]. To our knowledge, this is the first case reporting bilateral AACG caused by oxybutynin use.

This report serves to highlight the importance of constant vigilance when diagnosing and treating acute angle-closure glaucoma, particularly in cases with no clear risk factors and that are resistant to usual treatment. This case did not respond to the usual medical management for this condition and urgent bilateral laser peripheral iridotomies were needed.

## Conclusions

Bilateral AACG is a rare condition. It is considered an ophthalmological emergency and treatment delay would permanently jeopardize the patient’s vision. Different culprits have been reported and oxybutynin should be considered one of them. Careful review of the medical history and analysis of pertinent findings are crucial to ensure appropriate diagnosis and immediate treatment as required.
